# Low serum calcium is associated with higher long-term mortality in myocardial infarction patients from a population-based registry

**DOI:** 10.1038/s41598-021-81929-7

**Published:** 2021-01-28

**Authors:** Timo Schmitz, Christian Thilo, Jakob Linseisen, Margit Heier, Annette Peters, Bernhard Kuch, Christa Meisinger

**Affiliations:** 1 MONIKA/KORA Myocardial Infarction Registry, University Hospital of Augsburg, Augsburg, Germany; 2 LMU München at UNIKA-T Augsburg, Augsburg, Germany; 3 Department of Cardiology, University Hospital of Augsburg, Augsburg, Germany; 4 IRG Clinical Epidemiology, Helmholtz Zentrum München, Oberschleißheim, Germany; 5 KORA Study Centre, University Hospital of Augsburg, Augsburg, Germany; 6 Institute of Epidemiology, Helmholtz Zentrum München, Oberschleißheim, Germany; 7 German Center for Diabetes Research (DZD), Neuherberg, Germany; 8 Department of Internal Medicine, Hospital Nördlingen, Nördlingen, Germany

**Keywords:** Calcium and vitamin D, Cardiology

## Abstract

Calcium plays an essential role in physiology of the cardiovascular system. Aberrations from normal serum calcium levels are known to be associated with several cardiovascular diseases. Its possible role as a predictor for long-term mortality after acute myocardial infarction (AMI) is still uncertain. In this study, a total of 3732 patients (aged 25–74 years) with incident AMI surviving at least 28 days after AMI was included. The median follow-up time was 6.0 years. Admission total serum calcium levels were divided into quartiles. The Kaplan–Meier-Curve suggested a division of the follow up time in two different time periods. So, Cox regression models were calculated to assess association between admission serum calcium levels and all-cause long-term mortality with two observation periods: 28–2500 days and > 2500 days. The final model was adjusted for various comorbidities, clinical characteristics, in-hospital treatment and medication. The third quartile (normal-high Calcium levels) served as the reference group. The fully adjusted Cox-regression model shows significantly higher mortality risk for low serum calcium (quartile 1) within the timeframe 28–2500 days after the event (OR 1.53 [1.19–1.98]). The other groups did not differ significantly from each other. In the later observation period (from 2500 days until death or censoring) no more significant differences were seen between the four calcium quartiles. In summary, low serum calcium is an independent predictor of adverse outcome in the first 2500 days (about 7 years) after AMI. On later points in time this effect attenuates, so that no more significant differences can be observed.

## Introduction

Calcium is known to play a crucial role in pathophysiology of many diseases^1^. This is particularly true for the cardiovascular system and its diseases. Calcium is involved in the mechanism of vasoconstriction and therefore influences blood pressure. Furthermore, calcium ions are an essential part of the electrical conduction in the heart^2^. Disruptions in the calcium homeostasis can lead to serious conditions like malignant arrhythmia or cardiac arrest^2^. Previous studies suggest, that hypocalcaemia as well as hypercalcemia might be independent risk factors for in-hospital and short-term mortality after AMI^3–5^. However, only few studies examined associations between serum calcium levels and all-cause long-term mortality in AMI patients. Especially data from completely population-based registries with the possibility of multivariate adjustment for comorbidities, clinical characteristics and in-hospital treatment is scare. So the aim of this study was to investigate the association between admission serum calcium levels and long-term mortality in patients with incident AMI from a population-based registry.

## Methods

The underlying data for this research was collected by the KORA Myocardial Infarction Registry. It was established in 1985 as a part of the MONICA-project and since 1995 it operates within the KORA (Cooperative Health Research in the region of Augsburg) framework as KORA Myocardial Infarction Registry. The study area consists of the city of Augsburg, Germany, and the two adjacent counties of a total of approximately 650,000 inhabitants. All cases of fatal and non-fatal AMI are recorded, if the patients are between age 25 and 74 and have their primary residence in the study area. For this study, we considered all cases of patients, who survived the first 24 h after hospital admission. Trained study nurses carry out interviews using standardized questionnaire during the hospital stay. Further data collection is done by elaborating the patient´s medical files. In this way a large amount of data for each case of AMI is collected including information on sociodemographic characteristics, risk factors, comorbidities, diagnostics and treatment. Data on long-term survival is assessed regularly by mortality follow-ups and information derived from the competent registration and health offices. More detailed information on data collection is available in previous publications^6,7^.

For this study cases between January 1, 2000 and December 2008 were considered. Only patients with a first time myocardial infarction, who survived the first 28 days after the event, were included. Cases with missing information on calcium levels or missing information on relevant covariates were excluded.

Admission levels of total serum calcium were divided into quartiles: low admission calcium (0–2.18 mmol/L), normal-low admission calcium (2.18–2.29 mmol/L), normal-high admission calcium (2.30–2.40) and high admission calcium (from 2.41 mmol/). Serum levels of total calcium were measured by the laboratories of the participating hospital. All laboratories continuously performed internal quality control and met the official requirements. Maximum deviation of measured values can´t be specified due to varying measuring devices in different laboratories and changes over the study period of about 10 years.

Preexisting comorbidities such as diabetes, hyperlipidemia and hypertension as well as smoking status and typical chest pain symptoms at the event were determined during the interview and validated by chart review if possible. The admission ECG was evaluated by physicians. Each case was assigned to one of the following three groups: ST-elevation myocardial infarction (STEMI), Non-ST-evaluation myocardial infarction (NSTEMI) and bundle branch block.

As the kidney function has major influence on electrolyte levels, admission creatinine levels were used to calculate an estimated glomerular filtration rate (eGFR). Therefore, the recommended CKD-EPI-formula was used to calculate eGFR^8^. In accordance to the WHO classification of renal function impairment four groups were build: normal or slightly impaired renal function with an eGFR of more than 60 mL/min/1.73 m^2^, mild renal impairment when eGFR values were between 30 and 60 mL/min/1.73 m^2^, severe renal impairment with eGFR values lower than 30 ml/min/1.73 m^2^ and a group of unknown renal function due to missing creatinine values.

One common variable was created for any in-hospital revascularization (yes/no). This includes percutaneous coronary intervention (PCI), coronary artery bypass surgery and thrombolysis therapy.

For any in-hospital complication including cardiogenic shock, left ventricular decompensation, bradycardia, reinfarction, ventricular tachycardia and ventricular fibrillation, one variable was generated (yes/no).

One additional variable (yes/no) was created for all four evidence-based medications at discharge (EBM): antiplatelet agents, beta-blockers, angiotensin-converting enzyme inhibitors (ACEIs) or angiotensin-receptor blockers (ARBs) and statins. All four are considered standard therapy after AMI.

### Statistical analysis

Baseline characteristics and potential covariates were cross-tabulated with the four calcium groups. Categorical variables are presented as total number and percentages, continuous variables are described as median and interquartile range. To determine differences, Chi^2^ test for categorical variables and ANOVA (analysis of variance) for continuous variable were performed. P values < 0.05 are considered statistically significant.

For long time survival, we modeled three different COX regressions. The normal-high calcium group was chosen to be the reference group. The first model was calculated only for admission serum calcium group. The second model was adjusted for sex and age. A full model then was calculated including age, sex, renal function (eGFR), diabetes, hypertension, smoking status, hyperlipidemia, chest pain symptoms, STEMI/NSTEMI, any in-hospital complication, any Intervention (PCI, Bypass, lysis therapy), diuretics before AMI, calcium channels blockers before AMI, diuretics at discharge calcium channels blockers at discharge, all four evidence-based medications (EBMs). By hand-wise selection, potential covariates were taken into the full model if they had a significant log rank test (P-value < 0.05) or proved to have a significant impact in a Cox-regression model together with admission calcium (P-value < 0.05). Peak CKMB levels did not make a significant contribution to the model and consequently, this variable was not included in the fully adjusted COX-regression model. Furthermore, C-reactive protein (CRP), which was also initially considered as a covariate, didn´t reach significance in the Cox-regression model and was therefore not included in the final model.

The assumption of proportional hazards was checked by plotting the Schoenefeld residuals against time and looking for any visible correlation. Additionally, a test was performed to check for a significant correlation of the Schoenfeld residuals with time and consequently a violation of the proportional hazard assumption. This was the case for the admission calcium group, age, smoking status, any in-hospital revascularization and any in-hospital complication. A non-interaction stratification was performed for smoking status, any in-hospital complication and any in-hospital revascularization. That means that the Cox-model was stratified for these variables, but only one HR was calculated from the combination of both models. Considering the Kaplan–Meier curve for long term survival of the four Calcium groups, it was apparent, that at about 2500 days after the event the lines began to violate the proportional hazards assumption. To receive valid HRs for the admission calcium groups a time step function was implemented including two time periods: the first 2500 days after the event and the time from 2500 days until death or censoring time. The same step function was done for the age variable as well. In consequence, no variable showed significant correlation of the Schoenefeld residuals and time any more so that the proportional hazard assumption was given for every variable. Variance inflation factor (VIF) was used to assess multicollinearity among the independent variables.

The statistical analysis was performed by R version 3.6.1 (2019-07-05)^9^.

### Ethics approval and consent to participate

Data collection of the MONICA/KORA MI registry has been approved by the ethics committee of the Bavarian Medical Association (Bayerische Landesärztekammer) and the study was performed in accordance with the Declaration of Helsinki. All study participants have given written informed consent.

## Results

Between January 2000 and December 2008, a total of 6073 cases of patients with AMI, who survived the first 24 h after hospitalization, was recorded by the registry. Of those, 424 patients died within the first 28 days after infarction and were excluded. Of the remaining cases, 4514 patients had a first time myocardial infarction, the non-incidental cases were excluded. 782 cases were excluded due to missing values on long-term survival or relevant covariates. In the end, 3732 cases were included into further analysis. Median age was 59.9 (SD 9.8) years, and 24.5% of all patients were female (n = 913). The median follow-up time was 6.0 years.

Baseline characteristics of relevant covariates are displayed in Table 1. The four serum calcium groups significantly differed from each other in terms of age, presence of hyperlipidemia, typical chest pain symptoms, admission ECG, renal function (eGFR), admission troponin-I, calcium channel blockers at admission, application of PCI, thrombolysis, prescription of beta blockers, ACEIs/ARBs, statins, and diuretics at discharge.

**Table 1:** Baseline characteristics of AMI patients (n = 3732) by admission total serum calcium.

	Low calcium	Normal-low clacium	Normal-high calcium	High calcium	p-value*
Female	214 (22.8)	236 (22.7)	267 (26.6)	196 (26)	–
Age in years (mean + sd)	60.7 (9.5)	60.2 (9.9)	59.9 (9.7)	58.8 (10)	0.001
Total deaths (percentages): > 28–2500 days	152 (16,2%)	117 (11,3%)	106 (10,6%)	80 (10,6%)	0.00024
Total deaths (percentages): > 2500 days	8 (3.6%)	24 (6.5%)	32 (7.6%)	42 (10.8%)	0.0083
**Co-morbidities and risk factors at admission**					
Hypertension	715 (76.3)	782 (75.2)	745 (74.4)	559 (74.2)	0.722
Diabetes mellitus	277 (29.6)	295 (28.4)	287 (28.6)	209 (27.8)	0.869
Hyperlipidemia	579 (61.8)	745 (71.6)	706 (70.5)	581 (77.2)	< 0.0001
Current smoker	375 (40)	390 (37.5)	400 (39.9)	302 (40.1)	0.220
Ex-smoker	291 (31.1)	350 (33.7)	289 (28.8)	215 (28.6)	–
Never-smoker	271 (28.9)	300 (28.8)	313 (31.2)	236 (31.3)	–
**Clinical characteristics at admission**					
Typical chest pain symptoms	801 (85.5)	926 (89)	895 (89.3)	643 (85.4)	0.008
STEMI	326 (34.8)	393 (37.8)	410 (40.9)	319 (42.4)	0.006
NSTEMI	546 (58.3)	598 (57.5)	546 (54.5)	401 (53.3)	
Bundle branch block	65 (6.9)	49 (4.7)	46 (4.6)	33 (4.4)	
Any in-hospital complication	152 (16.2)	128 (12.3)	143 (14.3)	119 (15.8)	0.062
**Labaratory values at admission**					
eGFR < 30 mL/min/1.73 m^2^	31 (3.3)	7 (0.7)	12 (1.2)	9 (1.2)	< 0.0001
eGFR 30–60 mL/min/1.73 m^2^	106 (11.3)	74 (7.1)	69 (6.9)	37 (4.9)	–
eGFR 60 mL/min/1.73 m^2^	401 (42.8)	435 (41.8)	353 (35.2)	173 (23)	–
eGFR missing value	399 (42.6)	524 (50.4)	568 (56.7)	534 (70.9)	–
Admission troponin-I (ng/mL)	0.49 (0.1–2.99)	0.44 (0.1–2.88)	0.53 (0.12–3.46)	0.97 (0.16–5.785)	0.035
Peak CKMB levels	43 (14—111)	39 (15—107)	41 (15—101)	44 (17—104)	0.742
**Medication at admission**					
Calcium channel blockers	146 (15.6)	145 (13.9)	138 (13.8)	138 (18.3)	0.034
Diuretics	184 (19.6)	179 (17.2)	190 (19)	159 (21.1)	0.206
**In-hospital treatment**					
PCI	658 (70.2)	711 (68.4)	664 (66.3)	469 (62.3)	0.004
CABG	136 (14.5)	178 (17.1)	160 (15.9)	120 (15.9)	0.476
Thrombolysis	52 (5.5)	85 (8.2)	122 (12.1)	123 (16.3)	< 0.0001
Any revascularization therapy	787 (84)	897 (86.2)	847 (84.5)	624 (82.9)	0.245
**Medication at hospital discharge**					
All four EBMs	659 (70.3)	776 (74.6)	685 (68.4)	481 (63.9)	< 0.0001
Antiplatelet agents	904 (96.5)	1002 (96.3)	970 (96.8)	724 (96.1)	0.895
Beta-blockers	898 (95.8)	1014 (97.5)	947 (94.5)	702 (93.2)	0.0001
ACEIs/ARBs	775 (82.7)	890 (85.6)	814 (81.2)	590 (78.4)	0.001
Statins	836 (89.2)	940 (90.4)	884 (88.2)	639 (84.9)	0.003
Calcium channel blockers	124 (13.2)	132 (12.7)	121 (12.1)	101 (13.4)	0.827
Diuretics	498 (53.1)	522 (50.2)	503 (50.2)	323 (42.9)	0.0003

Categorical data presented as total numbers and % (proportion within each calcium group).

Numeric data presented as median and (IQR).

*Chi^2^ test was applied for categorical variables and ANOVA (analysis of variance) was applied for continuous variables. P values < 0.05 are considered statistically significant.

**Figure 1:**
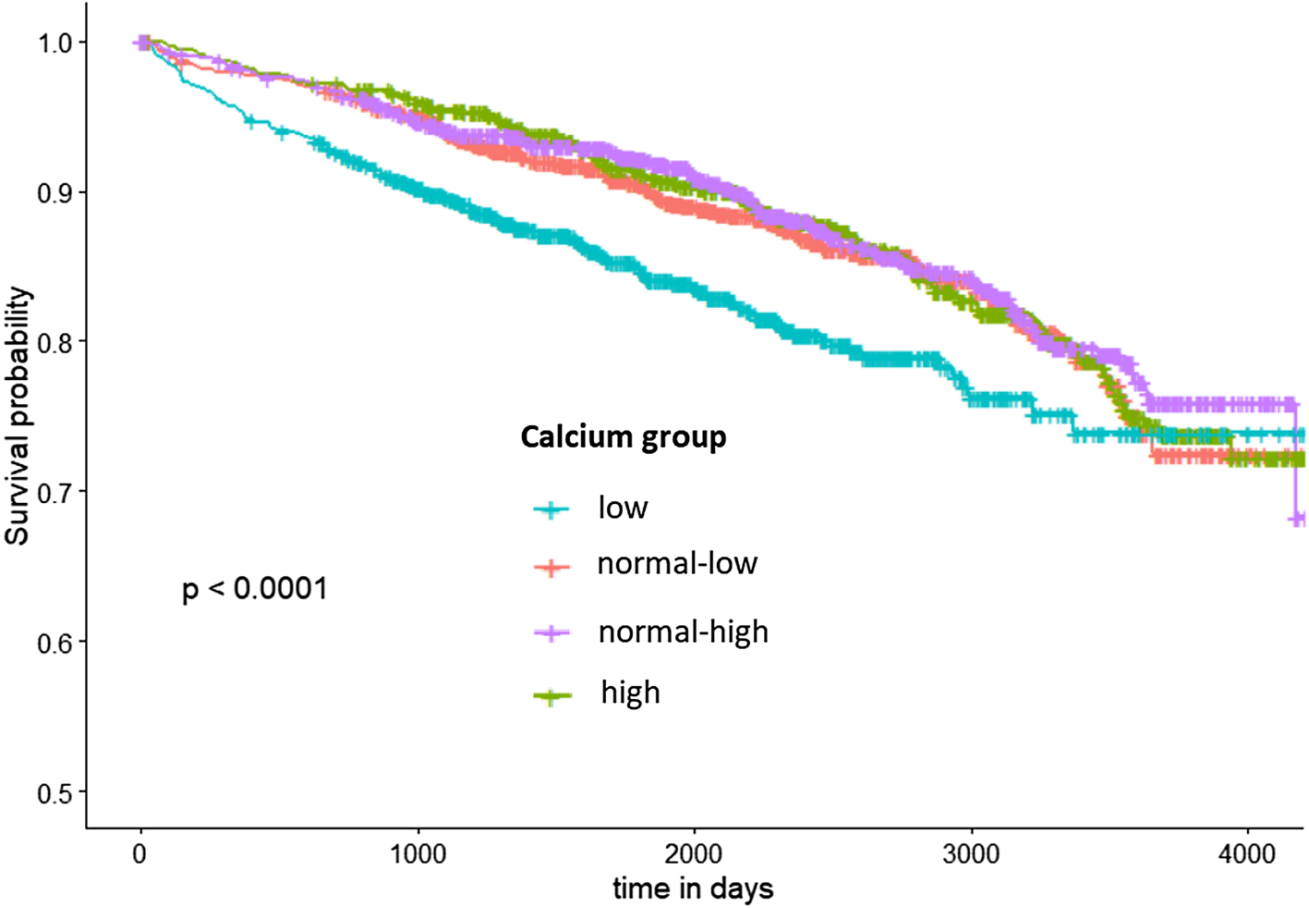
Kaplan–Meier Curve: long-term survival of AMI patients after 28 days by admission serum calcium concentration groups, figure generated with R version 3.6.1^9^.

Figure 1 shows the Kaplan–Meier curves for the four admission serum calcium groups. While the curves for normal-low group, normal-high group and high calcium group are pretty similar for the entire observation period, the low calcium group has a noticeably deviating graph. In the earlier stages low calcium is associated with higher all-cause mortality, but this trend can´t be observed for the later stages. Consequently, the follow-up time was divided in two time-periods, namely 28–2500 days and > 2500 days.

In the unadjusted Cox regression model low serum calcium has a significantly higher mortality from day 28 until day 2500 (HR: 1.76). Normal-low (HR: 1.10) and high serum calcium (HR: 0.96) on the other hand do not vary significantly from the reference group (normal-high) in the first time period. In the second time period from day 2500 no calcium group differed significantly from the reference group (see Table 2).

**Table 2:** COX-Regression models by calcium groups; an unadjusted model, a model adjusted for sex and age and a fully adjusted model.

	28–2500 days		> 2500 days	
	HR (95% CI)	P value	HR (95% CI)	P value
**Unadjusted model**				
Calcium low	1.76 (1.37–2.25)	< 0.0001	0.68 (0.31–1.48)	0.331
Calcium normal-low	1.10 (0.84–1.43)	0.487	1.05 (0.62–1.78)	0.871
Calcium normal-high	1 (Ref)	–	1 (Ref)	–
Calcium high	0.96 (0.72–1.29)	0.792	1.27 (0.79–2.00)	0.313
**Model adjusted for sex and age**				
Calcium low	1.71 (1.33–2.19)	< 0.0001	0.70 (0.32–1.52)	0.363
Calcium normal-low	1.08 (0.83–1.40)	0.5778	1.02 (0.60–1.74)	0.931
Calcium normal-high	1 (Ref)	–	1 (Ref)	–
Calcium high	1.04 (0.77–1.38)	0.816	1.36 (0.86–2.15)	0.193
**Fully adjusted model**^**a**^				
Calcium low	1.53 (1.19–1.98)	0.000936	0.65 (0.30–1.42)	0.284
Calcium normal-low	1.18 (0.91–1.54)	0.221	1.01 (0.59–1.72)	0.981
Calcium normal-high	1 (Ref)	–	1 (Ref)	–
Calcium high	0.99 (0.74–1.33)	0.954	1.32 (0.83–2.10)	0.234

Two observational periods were distinguished: from 28 to 2500 days after incidental AMI and more than 2500 days after incidental AMI.

^a^Adjusted for age, sex, renal function (eGFR), diabetes, hypertension, smoking status, hyperlipidemia, chest pain symptoms, STEMI/NSTEMI, any in-hospital complication, any intervention (PCI, bypass, lysis therapy), diuretics before AMI, calcium channels blockers before AMI, diuretics at discharge, calcium channels blockers at discharge, all four evidence-based medications (EBMs).

Adjusting for sex and age did not result in a notable difference for the HR’s (see Table 2). Low serum calcium remains being significantly associated with higher mortality in the first time period (HR: 1.71), but not so in the second time period (HR: 0.70). Normal-low and high serum Calcium did not have a significantly different mortality risk for any of the two time periods.

The final model was further adjusted for age, sex, renal function (eGFR), diabetes, hypertension, smoking status, hyperlipidemia, chest pain symptoms, STEMI/NSTEMI, any in-hospital complication, any intervention (PCI, bypass, lysis therapy), diuretics before AMI, calcium channels blockers before AMI, diuretics at discharge, calcium channels blockers at discharge, all four evidence-based medications (EBMs). The result still showed a significant higher mortality for the low calcium group in the first time period (HR: 1.53). For the second time period, low serum calcium as well as normal-low and high serum calcium did not have significantly different mortality risks compared to the reference group (see Table 2).

Displayed in the “Supplementary material S1”, further COX-regression models were calculated. Firstly, three models (unadjusted, adjusted for sex and age, fully adjusted) without the distinction into the two time-periods were conducted. In this analysis, low serum calcium was still significantly associated with poorer long-term outcome in the fully adjusted model resulting in a HR of 1.5 (see Supplementary Table 1). Secondly, COX-regression models (unadjusted, adjusted for sex and age, fully adjusted) were conducted with continuous serum total calcium values. Increasing serum calcium levels were significantly inversely associated with long-term mortality in the first time period (HR per 1 mmol/L increase in serum calcium: 0.92; 95% CI 0.86–0.99), but not so in the second time period or in the total time period (see Supplementary Table 2).

## Discussion

Low serum calcium is significantly associated with higher all-cause mortality in the timeframe 28–2500 days after AMI compared to the reference group (normal-high calcium). There is no significant difference between the other calcium groups in this time period. All four calcium groups did not differ from each other in the timeframe > 2500 days after the event. The division of the follow-up time in those two time-periods was done post-hoc based on Kaplan–Meier-curve and Scheonfeld analysis.

Prior studies examined in-hospital and short-term mortality for admission calcium levels after AMI. They found increased mortality especially for decreased admission serum calcium^3–5^, but also for hypercalcemia^4^. We excluded patients, who died within the first 28 days after the event in order to concentrate on long term mortality exclusively. Because our data was obtained from a multicenter survey and the admission serum calcium levels were measured by different laboratories, we decided to build quartiles to categorize the cases into four calcium groups (low, normal-low, normal-high and high), as suggested by other studies. For the present study, calcium status was determined by total serum calcium measurements, a method which is widely used to assess calcium status of patients in daily clinical practice. About 50% of total serum calcium is present in form of ionized calcium^10^; only ionized and free, that is not albumin-bound serum calcium is biologically active^10^. Thus, assessment of the calcium status can also be based on the measurement of ionized calcium. Particularly in patients with suspected calcium disorders, calcium status estimations can differ from each other when either based on total serum calcium or ionized serum calcium^11^. This especially applies to certain conditions like hypoalbuminemia, which is why total serum calcium levels can be corrected for albumin concentrations^12^. Nevertheless, recent studies questioned, whether albumin adjustment of total calcium does improve the estimation of calcium status^13–15^. Some prior studies on association of calcium levels and outcome of cardiovascular diseases based their analyses on ionized calcium concentrations which causes a limited comparability with this study^16,17^. However, the majority of the previous studies also based their results on total serum calcium leading to a good comparability with the results of the present study.

Total serum calcium levels can be influenced by many parameters. One important factor is impaired renal function, which can affect serum calcium^18,19^. Certain medications like diuretics and calcium channels blockers might influence serum calcium levels as well^20–22^. Therefore, they were considered as potential covariates and were kept in the model if they reached the significance criterion. Accordingly, the final model was adjusted for renal function and intake of diuretics and calcium channel blockers before the event.

Serum calcium is known to be a predictor of long-term mortality in different cardiovascular diseases^23,24^. There are previous studies, which examined associations between serum calcium levels and long-term mortality in patients with coronary heart diseases and after AMI^25–28^. Jiang et al. investigated 192 patients with ST-Elevation AMI and divided them into a normal calcium group and a hypocalcemia group. In a multivariate logistic regression for survival after 150 days, they found the hypocalcemia group to have significantly worse mid-term survival than the normal calcium group^25^. A further study found similar results^26^. Xingbo Gu et al. compared mid-term mortality of 2594 patients with acute coronary syndrome according to serum calcium. They split up the cases in quartiles as in the present study. Multivariate Cox-regression with a median follow-up period of 21.8 months showed significantly higher mortality among the lowest calcium group and no significant differences for the other three groups. This is very close to what we found for the first time period after the event (28–2500 days). In our study, this effect starts to weaken over time and no more significant differences can be found for the later observation time. It seems plausible, that a laboratory value has a more reliable predictive value in the nearer future and that its predictive value weakens as time goes on.

Researchers from a Chinese study presented similar results for patients with established coronary heart diseases^27^. The quartile of patients with the lowest serum calcium levels had the highest risk of long-term mortality (median follow-up: 4.9 years). Higher serum calcium levels did not correlate with higher mortality.

Another study from 2012 examined associations between baseline calcium levels and risk of cardiovascular and all-cause mortality in a population with stable coronary heart disease. 1206 patients were followed up for 8 years. The quartile of patients with the highest serum calcium levels had the highest risk of all-cause mortality compared to the other calcium groups^28^. This is in a way contrary to what we found. Though, comparability is limited due to different time points of measurement of serum calcium levels (at time at AMI versus a stable state of coronary heart disease).

Nevertheless, the exact mechanisms are unclear how decreased admission calcium levels lead to a higher long-term mortality after AMI. Blood calcium levels and intracellular calcium homoeostasis are regulated precisely and even small deviations can lead to organic malfunction especially in cardiac electrophysiological processes^29–31^. Several studies found associations between decreased serum calcium levels and some cardiovascular risk factors such as hypertension^32,33^, diabetes mellitus type 2^34^, smoking^35^ or left ventricular systolic dysfunction^36^. This might contribute to a higher cardiovascular and all-cause mortality in the low calcium group. Nevertheless, we adjusted the final COX regression model for those comorbidities and low serum calcium was still a significant risk factor for mortality in the time between 28 and 2500 days after AMI. Nonetheless we must consider insufficient information on these comorbidities and therefore the possibility of residual confounding. Furthermore, we might not have considered all relevant confounders which can be associated with decrease serum calcium. An important possible confounder in this regard might be the presence of a malignant disease. A study from China with 25,000 cancer patients revealed that 26.7% of them had hypocalcemia^37^. The presence of hypocalcemia was also associated with a higher in-hospital-mortality compared to cancer patients without any electrolyte disorders^37^. Thongprayoon et al. examined the association of long-term mortality among hospitalized patients with various admission serum ionized calcium levels and found that hypocalcemia was significantly associated with higher long-term mortality even after multivariate adjustment^38^. They suggested, that ionized serum calcium could be viewed as a sick index or marker of disease severity.

Furthermore, Thongprayoon et al. found that patients with low serum ionized calcium carried an increased risk of ventricular arrhythmia^38^. It is also known, that low admission serum calcium levels are independently associated with an increased risk of sudden cardiac arrest^39^. One decisive factor for this could be a prolonged QT interval, which is known to be a risk factor for sudden cardiac death^40^. Some studies indeed found an association between low serum calcium and prolonged QT interval time^41,42^. Furthermore, calcium supplementation seems to be effective for shortening repolarization intervals^42^. Nevertheless, some limitations to our study in this regard must be considered. Firstly, no information on patients QT interval time was available, so it is not possible to determine, weather low serum calcium is indeed associated with prolonged QT intervals in patients included in this study. Secondly, since all-cause mortality was the primary outcome of the study and no further information on the cause of death was available, we are no able to differentiate between mortality caused by sudden cardiac arrests, mortality caused by other cardiovascular diseases and non-cardiovascular mortality caused by other diseases.

As a consequence, we can only speculate, on whether hypocalcemic patients with AMI would benefit from calcium supplementation in a long term. More research is needed in this regard.

### Strengths and limitations

There are several strengths of this study. First, the high number of cases from a population-based registry with consecutive enrollment avoids selection bias. The large amount of collected data on relevant covariates such as sociodemographic information, risk factors, comorbidities and information on in-hospital complications and treatment provides the opportunity for extensive adjustments. With a median follow-up time of 6.0 years the observation period after the event is quite long.

Nevertheless, the following limitations to our study must be mentioned. Since only patients up to 74 years were included, results cannot necessarily be applied to older patients. Moreover, the results may not be generalized to all ethnic groups since no information on ethnicity was available. Since this is a multicenter study, admission serum calcium levels were measured by different laboratories, which may cause some bias. Moreover, no information on ionized calcium concentrations and serum albumin concentrations was available. As only free and not albumin-bound calcium is physiologically active, this must be considered as a further limitation of this study. Furthermore, the outcome of the study was all-cause death and no information on the cause of death was available for this study. Also, no data was collected on treatment of abnormal serum calcium levels. Finally, we might not have considered all relevant confounders and cannot exclude possible reverse causation.

## Conclusion

Low serum total calcium levels at hospital admission are independently associated with increased mortality after incident AMI for the time period of 28–2500 days after the event. On the time period past 2500 days, no more significant difference in long-term mortality is seen among the four admission serum calcium groups. It is unclear though, whether patients with hypocalcemia would benefit from calcium supplementation for improved long-term mortality. Further studies on this subject are needed.

## Supplementary Information

Supplementary Information.

## Data Availability

The data will not be shared. Due to restrictions from Helmholtz Zentrum München, data are available upon request for any researcher based on a standard agreement on data provision within the KORA Research Platform.
