# Supplementary material for: Low serum calcium is associated with higher long-term mortality in myocardial infarction patients from a population-based registry
Source: Sci Rep. 2021 Jan 28;11:2476. doi: 10.1038/s41598-021-81929-7 (PMC7843683; doi:10.1038/s41598-021-81929-7)
Supplement: Supplementary file 1 — Supplementary Information. [file 41598_2021_81929_MOESM1_ESM.docx]

***Low serum calcium is associated with higher long-term mortality in myocardial infarction patients from a population-based registry***

Timo Schmitz^1,2^, Christian Thilo^3^, Jakob Linseisen^2,4^, Margit Heier^5,6^, Annette Peters^6,7^, Bernhard Kuch^8^, Christa Meisinger^2,4^

^1^ MONIKA/KORA Myocardial Infarction Registry, University Hospital of Augsburg; ^2^Chair of Epidemiology, LMU München at UNIKA-T Augsburg; ^3^University Hospital of Augsburg, Department of Cardiology; ^4^ IRG Clinical Epidemiology, Helmholtz Zentrum München; ^5^University Hospital of Augsburg, KORA Study Centre, ^6^Institute of Epidemiology, Helmholtz Zentrum München; ^7^German Center for Diabetes Research (DZD) Neuherberg Germany, ^8^Department of Internal Medicine, Hospital Nördlingen, Germany

Correspondence to: Timo Schmitz, t.schmitz@unika-t.de

***Supplementary material***

**Table 1: COX-Regression models for the association between calcium groups and long-term mortality for one time period including an unadjusted model, a model adjusted for sex and age and a fully adjusted model.**

|  | ***Unadjusted model*** | | ***Adjusted for sex and age*** | | ***Fully adjusted model**** | |
| --- | --- | --- | --- | --- | --- | --- |
|  | ***HR***  ***(95% CI)*** | ***p Value*** | ***HR***  ***(95% CI)*** | ***p Value*** | ***HR***  ***(95% CI)*** | ***p Value*** |
| ***Calcium low*** | 1.6  (1.3 - 2.0) | < 0.001 | 1.6  (1.3 - 2.0) | < 0.001 | 1.5  (1.2 - 1.9) | 0.0012 |
| ***Calcium normal-low*** | 1.1  (0.9 - 1.4) | 0.494 | 1.07  (0.8 - 1.3) | 0.594 | 1.2  (0.9 - 1.5) | 0.201 |
| ***Calcium normal-high*** | 1 (Ref) | - | 1 (Ref) | - | 1 (Ref) | - |
| ***Calcium high*** | 1.1  (0.8 - 1.3) | 0.688 | 1.13  (0.9 -1.4) | 0.326 | 1..1  (0.9 - 1.4) | 0.524 |

*adjusted for age, sex, renal function (eGFR), diabetes, hypertension, smoking status, hyperlipidemia, chest pain symptoms, STEMI/NSTEMI, any in-hospital complication, any intervention (PCI, bypass, lysis therapy), diuretics before AMI, calcium channels blockers before AMI, diuretics at discharge, calcium channels blockers at discharge, all four evidence-based medications (EBMs)

**Table 2: COX-Regression models for the association between continuous total serum calcium levels and long-term mortality for the two time periods and the whole time period.**

|  | ***28 -2500 day*** | | ***> 2500 days*** | | ***one time period*** | |
| --- | --- | --- | --- | --- | --- | --- |
|  | ***HR***  ***(95% CI)*** | ***p Value*** | ***HR***  ***(95% CI)*** | ***p Value*** | ***HR***  ***(95% CI)*** | ***p Value*** |
| ***Unadjusted model*** | | | | | | |
| *Calcium continuous* | 0.93  (0.86 - 0.99) | 0.0345 | 1.02  (0.93 - 1.13) | 0.6436 | 0.95  (0.90 - 1.01) | 0.0922 |
| ***Adjusted for sex and age*** | | | | | | |
| *Calcium continuous* | 0.93  (0.87 - 0.99) | 0.0377 | 1.02  (0.92 - 1.12) | 0.7280 | 0.9528  0.90 - 1.01) | 0.0896 |
| ***Fully adjusted model**** | | | | | | |
| *Calcium continuous* | 0.92  (0.86 - 0.99) | 0.022625 | 1.03  (0.93 - 1.14) | 0.561903 | 0.95  (0.90 - 1.00) | 0.069865 |

*adjusted for age, sex, renal function (eGFR), diabetes, hypertension, smoking status, hyperlipidemia, chest pain symptoms, STEMI/NSTEMI, any in-hospital complication, any intervention (PCI, bypass, lysis therapy), diuretics before AMI, calcium channels blockers before AMI, diuretics at discharge, calcium channels blockers at discharge, all four evidence-based medications (EBMs)
